# Motivation states to move, be physically active and sedentary vary like circadian rhythms and are associated with affect and arousal

**DOI:** 10.3389/fspor.2023.1094288

**Published:** 2023-04-18

**Authors:** Christopher J. Budnick, Matthew Stults-Kolehmainen, Cyrus Dadina, John B. Bartholomew, Daniel Boullosa, Garret I. Ash, Rajita Sinha, Miguel Blacutt, Adrian Haughton, Tom Lu

**Affiliations:** ^1^ Department of Psychology, Southern Connecticut State University, New Haven, CT, United States; ^2^ Division of Digestive Health, Yale New Haven Hospital, New Haven, CT, United States; ^3^ Department of Biobehavioral Sciences, Teachers College—Columbia University, New York, NY, United States; ^4^ Science Research Program, Dobbs Ferry High School, Dobbs Ferry, NY, United States; ^5^ Department of Kinesiology and Health Education, The University of Texas at Austin, Austin, TX, United States; ^6^ Faculty of Physical Activity and Sports Sciences, Universidad de León, León, Spain; ^7^ College of Healthcare Sciences, James Cook University, Townsville, Australia; ^8^ Graduate Program in Movement Sciences, Integrated Institute of Health, Federal University of Mato Grosso do Sul, Campo Grande, Brazil; ^9^ Center for Pain, Research, Informatics, Medical Comorbidities and Education Center (PRIME), VA Connecticut Healthcare System, West Haven, CT, United States; ^10^ Center for Medical Informatics, Yale School of Medicine, New Haven, CT, United States; ^11^ Department of Psychiatry, Yale Medical School, New Haven, CT, United States; ^12^ Department of Psychology, University of Notre Dame, Notre Dame, IN, United States; ^13^ Patel College of Osteopathic Medicine, Nova Southeastern University, Fort Lauderdale, FL, United States; ^14^ Department of Mathematics and Statistics, Texas Tech University, Lubbock, TX, United States

**Keywords:** affectively charged, motivation states, affect, arousal, exercise, physical activity, sedentary activity, body position, sleep

## Abstract

**Introduction:**

Motivation to be physically active and sedentary is a transient state that varies in response to previous behavior. It is not known: (a) if motivational states vary from morning to evening, (b) if they are related to feeling states (arousal/hedonic tone), and (c) whether they predict current behavior and intentions. The primary purpose of this study was to determine if motivation states vary across the day and in what pattern. Thirty adults from the United States were recruited from Amazon MTurk.

**Methods:**

Participants completed 6 identical online surveys each day for 8 days beginning after waking and every 2–3 h thereafter until bedtime. Participants completed: (a) the CRAVE scale (Right now version) to measure motivation states for Move and Rest, (b) Feeling Scale, (c) Felt Arousal Scale, and (d) surveys about current movement behavior (e.g., currently sitting, standing, laying down) and intentions for exercise and sleep. Of these, 21 participants (mean age 37.7 y; 52.4% female) had complete and valid data.

**Results:**

Visual inspection of data determined that: a) motivation states varied widely across the day, and b) most participants had a single wave cycle each day. Hierarchical linear modelling revealed that there were significant linear and quadratic time trends for both Move and Rest. Move peaked near 1500 h when Rest was at its nadir. Cosinor analysis determined that the functional waveform was circadian for Move for 81% of participants and 62% for Rest. Pleasure/displeasure and arousal independently predicted motivation states (all *p*'s < .001), but arousal had an association twice as large. Eating, exercise and sleep behaviors, especially those over 2 h before assessment, predicted current motivation states. Move-motivation predicted current body position (e.g., laying down, sitting, walking) and intentions for exercise and sleep more consistently than rest, with the strongest prediction of behaviors planned for the next 30 min.

**Discussion:**

While these data must be replicated with a larger sample, results suggest that motivation states to be active or sedentary have a circadian waveform for most people and influence future behavioral intentions. These novel results highlight the need to rethink the traditional approaches typically utilized to increase physical activity levels.

## Introduction

Levels of physical activity remain low and levels of sedentarism remain high despite substantial efforts to improve these behaviors on a national and global scale (1, 2). Sophisticated new models, such as the Affect and Health Behavior Framework (AHBF) (3, 4), Affective Reflective Theory of Physical Inactivity (ART) (5), the dual process model from Conroy (6), and the behavior change wheel (7) have all identified impulses and motivation states as potential targets for intervention. This is in line with the Research Domain Criteria (RDoC) from the National Institute of Mental Health that has prioritized identifying behavioral “elements” for further exploration (8). In this vein, the WANT model (Wants and Aversions for Neuromuscular Tasks) was recently developed to understand how motivation states for movement and sedentarism operate (9). This model indicates that they work loosely and asynchronously. For instance, one may be high or low in motivation for both physical activity and rest simultaneously, or one may have shifting motivation for movement but not rest. These changes which facilitate flexible and adaptive behavior in response to stressful situations (9). Furthermore, using the CRAVE scale (Cravings for Rest and Volitional Energy Expenditure) (10), it has been determined that motivation states to move and rest morph quickly in response to a variety of stimuli and situations, such as exercise or periods of sitting, with effect sizes in the moderate to large range (10–12).

A key question currently centers on how motivation states vary across the day and the pattern of that variation—linear, curvilinear, or random/chaotic. Motivation to move and rest might follow a circadian pattern (13), which may have a stronger level of influence on behavior than many other factors. These assertions are typically based on observations of rodents and other animals (13), but little is known about daily fluctuations in human motivation to move or rest. The primary source of information comes from clinical populations, including those with Restless Leg Syndrome (RLS), which demonstrate altered patterns of urges to move with a circadian pattern peaking just after bedtime (14, 15). In fact, the urge to move is the defining feature of this disorder. In terms of physical activity itself, there have been recent calls to understand movement and sedentarism from a 24-hour activity perspective (16). These behaviors appear to have diurnal variation for most people (17) with the majority of adults (ages 18–60 y) having a relatively consistent pattern of activity from 10am to 6pm and of rest typically occurring from 11pm to 6am (18, 19). A recent qualitative study with focus groups with 17 college honors students found that a major theme surrounding motivation states was “change and stability”. Some participants indicated fluctuations in the desire to move and rest on a moment by moment, hourly, and daily level (12), which was partially validated with quantitative data collected pre- and post-interviews.

Alternatively, changes in motivation may be due to more random processes related to shifting conditions. Resnicow (20) argued that processes of change in motivation are chaotic. He has argued for a more quantum perspective of behavior change, and suggested that “motivation arrives as opposed to being planned” (21), being often akin to a randomly-occurring epiphany. They may also happen when certain tipping points are reached, and this has been hypothesized by Inzlicht (22) in his assertion that motivation changes in conditions of feeling deprived or overly fatigued. A recent paper attempted to reconcile these various perspectives and subsume them within the construct of motivational drive (23). It is possible that all these processes are at play, and they work in tandem and not necessarily in direct opposition. Unfortunately, there are few data in humans to make any meaningful conclusions about how motivation to be physically active trends over time.

Another pressing issue is that motivation states have rarely been linked concretely to future behavior *in healthy populations*. Until such a link can be firmly established, there is limited usefulness for the concept of highly transient motivation states for physical activity. In clinical populations, such as those with RLS, anorexia nervosa and akathisia, the connection between urges to move and subsequent behavior is well established (14, 15, 24, 25). However, in healthy populations, where there are less bothersome sensations, the link has been largely ignored or even hypothesized to not exist (26). Despite this large gap in knowledge, there are data to support the idea that motivation states co-occur and precede behavior if there are no barriers for subsequent behavior. For instance, qualitative interviews identified that motivation states are the result of previous behaviors and also result in subsequent activity, especially when motivation is very strong, like an urge or craving to work out after having been inactive for a prolonged period of time (12). Further advancement in this area is sorely needed.

Unresolved at this time is whether motivation states are most closely aligned with factors like affect and emotion or to cognitive processes (e.g., deliberation, reflection) and more stable goals. Williams and Bohlen (26) opined that reflection is the primary component of desires for physical activity and exercise, further arguing against the idea that desires for activity might be hedonic or appetitive in nature. Nonetheless, it seems clear that desires and urges to move and rest may also be instigated by and related to a variety of feeling and emotive states, such as elation (9, 27, 28). This is further supported by qualitative data (12) and various models of emotion and motivation for physical activity, such as the AHBF (3, 4), which predict that motivation states are downstream byproducts of transient affect/feeling states. This has a long precedent, perhaps starting with Festinger and the idea that psychological dissonance is a motivation state in which people make efforts to reduce tension (29).

**Figure 1:**
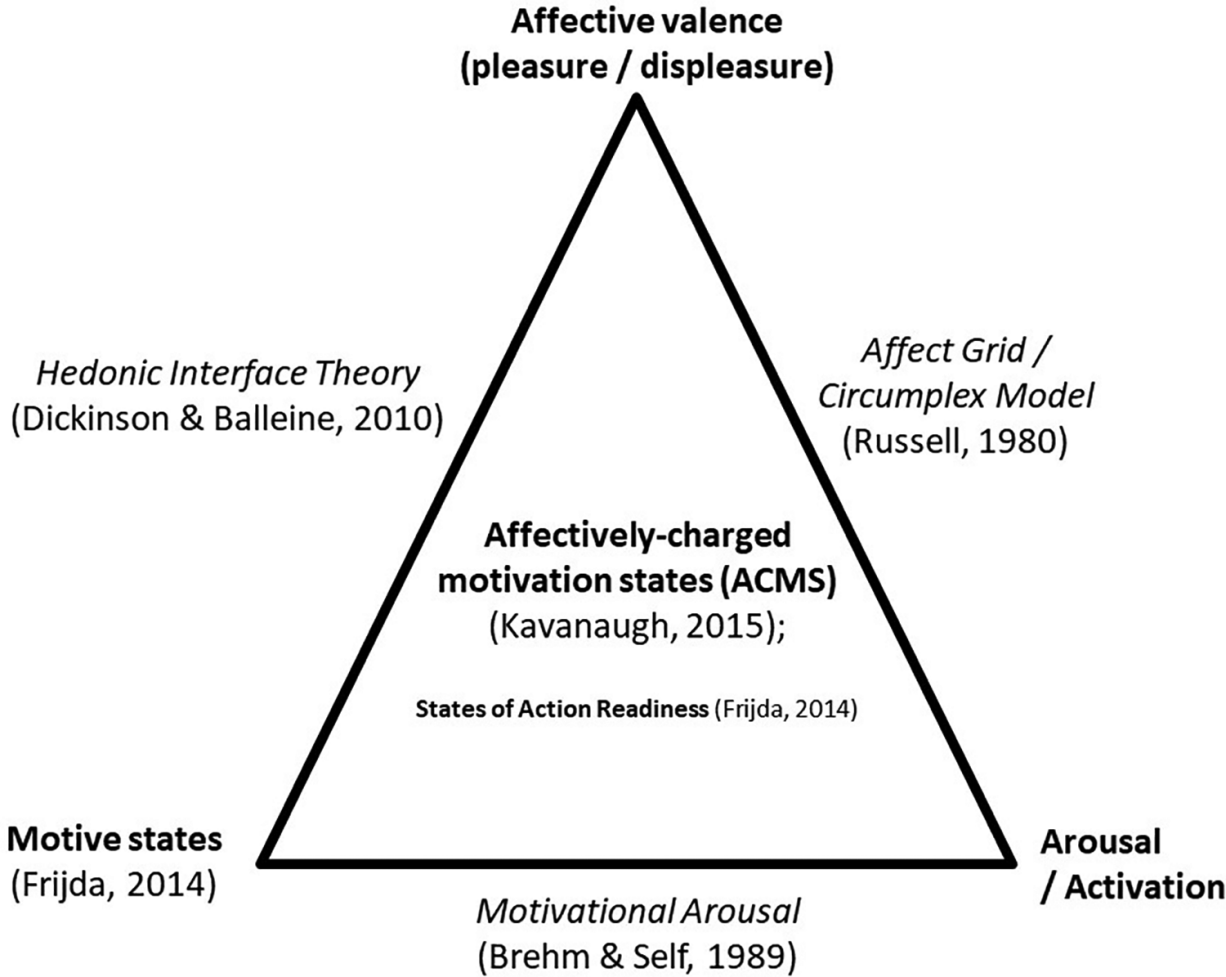
Theories of affect and motivation. In this illustration, affectively-charged motivation states (ACMS) (30) are the end product of interactions between the motive state (37), affective valence, and arousal (38). ACMS is a construct distinct but related to the ideas of motivational arousal (36), affect and the hedonic interface (39). A similar concept comes from Frijda (37) (States of Action Readiness), which proposes how ACMS result in motivated behavior.

More recently, Kavanaugh (30) coined the term “affectively-charged motivation states” (ACMS) to typify motivation states that are felt with a negative or positive tension. For instance, when indoors and cooped up for long periods, one may feel antsy or fidgety and have a “pressing readiness” to move and be active (9). In response to pleasant music at a high beat rate, one might feel compelled to move the body, which is called “groove” (31). Taylor and colleagues (32) have argued that pleasure/displeasure and arousal/activation are foundational to motivation for activity and perhaps more so than reflective factors. They stated, “Physiological responses to exercise and their generalized core affective labels (i.e., states that vary simply on pleasantness and activation) are motivationally salient because they form the basis of desires that are often contrary to valued goals. Indeed, the central purpose of affect associated with afferent bodily signals is to motivate action” (33–35). In contrast to perspectives that focus mainly on hedonic valence, Brehm and Self (36) have focused on the interface between motivation states and arousal/activation in the prediction of effort. Their concept of the *momentary magnitude of motivational arousal* (MMMA) accounts for both the motive and the amount of effort a person is able and willing to expend on a task. [Interrelations between motivation, hedonic valence, and arousal are demonstrated in Figure 1.] Currently, no data link the experience of pleasure/displeasure and arousal/activation with motivation states for movement and sedentarism. However, Stults-Kolehmainen and colleagues (10) did find small to moderate associations with energy and fatigue, which indicates that such associations likely exist.

To address the gaps in the literature discussed above, we used an Ecological Momentary Assessment approach (40), which is designed to capture snapshots throughout the day—in this case, urges to move and rest, arousal, affect, and behavioral intentions. This approach captures inter-individual variability and dynamic patterns of change.

We focused on the following research questions:

1. Do movement and sedentary wants/desires vary across the day? If so, what is the pattern of change?
2. Are there associations with pleasure and arousal, and do these interact?
3. Do previous behaviors impact these wants/desires?
4. Are these wants/desires associated with: (a) current body position (i.e., at the moment of inquiry, such as lying down, sitting, standing, et cetera), (b) current activities (i.e., eating, exercise, and sleep), and (c) intentions for health behaviors over the next few hours?

We hypothesized that there is a high degree of variability throughout the day but made no specific hypothesis for how those changes might manifest. We also hypothesized that previous behaviors would impact motivation states, and in turn, motivation states would be associated with current behaviors and intentions to be active. We lastly hypothesized that there would be an association between feeling states (including hedonic valence and arousal) and motivation states.

## Methods and materials

### Participants

Participants were 21 adults residing in the USA (mean age 37.7 y; 52.4% female, 29% people of color) who had complete and valid data.

### Procedures

#### Subject recruitment

Thirty participants were recruited through MTurk, Amazon's crowdsourcing platform. To complete the assignment, participants had to reside in the USA and be at least 18 years of age for reasons of consent. The data collected on MTurk included participants' race, gender, time zone, state/region of residence, and typical wake up time and bedtime. The MTurk assignment then directed the participants to an informed consent on Google Forms, which included a link to a downloadable informed consent document, as well as a version that could be read on the form itself. Upon fully completing the study according to terms in the consent, participants were awarded $50 USD (see below).

#### Data collection

After submission of informed consent, participants were promptly emailed regarding when their first survey would be and further instructions on how the study would be conducted. This email included their own link to the main surveys on SurveyMonkey, where two types of surveys were given.

A. The “PAST WEEK” type had two sets of motivation states questions:
  a. one set asking about motivation states “in the past week”, and
  b. one set asking about motivation states while the participant took the survey (“right now”).
  c. It also asked about Felt Arousal and Feeling “in the past week” and “right now”.
B. The “NOW” type survey only consisted of questions pertaining to motivation and affective states at the current moment (“right now”).

Both surveys contained the 10 additional closed-ended questions asking about felt arousal (current), hedonic valence (current), sleep, eating, et cetera. Each participant took the first survey type (A) at the beginning and end of the study. Participants were instructed to take the second survey type (B) 6 times per day for 8 days and were encouraged to take the survey once after waking up, once before going to sleep, and to spread the other four surveys apart throughout the day as much as possible. An Amazon Web Services EC2 instance was used as an email bot to remind participants to take surveys. Each participant was emailed six times throughout the day with their survey link and a reminder to spread the surveys apart and to take one after waking up and one before going to bed.

To be deemed eligible for the $50 payout, certain standards had to be met by each participant: (1) no less than 45 surveys submitted, (2) both “past week” CRAVE surveys submitted (Type A), (3) each survey took at least 30 s to complete (45 for Type A surveys), (4) no less than 3 surveys were submitted on a particular day, and (5) surveys were spread out across the day, i.e., surveys should not be submitted more than once in a particular 1-hour period. We chose the benchmark of 45 surveys because this would result in between 5 and 6 surveys per day. This would ensure that we received observations throughout each day for all 8 days.

All data was collected between July 2nd, 2021, and July 11th, 2021, with the majority of data collected between July 2nd, 2021, and July 9th, 2021. Around 1,400 surveys were collected, and 1,031 surveys were used for the study from 21 participants with complete and valid data. Participants submitted an average of 49.1 surveys per person (SD = 1.41, range = 46–51).

#### Instrumentation

*CRAVE scale*: Levels of motivation states to move and rest were self-recorded and submitted by participants using the Cravings for Rest and Volitional Energy Expenditure (CRAVE) Scale, a 13-item questionnaire consisting of statements regarding physical activity and sedentarism attached to 11-point Likert items. A subset of five items regard physical activity, e.g.,: “At this very moment, I want/desire to *move my body*”. Another subset of five items regard sedentarism, e.g.,: “At this very moment, I want/desire to *just sit down*”. The last three items are filler items that are not used for analysis. For each item, a participant would assign a number from zero to ten showing their agreement with the statement at the moment of taking the survey (“right now”). Scores for both subscales range from 0 to 50 with very high scores theoretically representing strong urges or cravings to move or rest. Participants also completed the “past week” version of the scale twice, which retrospectively assessed motivation states for the week before the study and the week during the study. These scales have excellent psychometric properties, as assessed over a series of 6 studies (10, 12). For the remainder of this paper “Move-NOW” and “Rest-NOW” refer to scores of the right-now version of the CRAVE scale. Concordantly, the terms “Move-WEEK” and “Rest-WEEK” refer to the scores of the CRAVE scale that assess motivation retrospectively over the past week.

*Feeling Scale* (*FS*): Affective valence (pleasure/displeasure), as conceptualized from the Circumplex Model, was recorded with the Feeling Scale (41). This is a single-item, 11-point bi-polar measure ranging from −5 (*very bad*) to +5 (*very good*); 0 represented *neutral*. The FS exhibits correlations ranging from .52 to .88 with the valence scale of the Self-Assessment Manikin (SAM) (42); and from .41 to .59 with the valence scale of the Affect Grid (AG) (38). Affective valence is an effective measure of pleasure/displeasure during exercise (43, 44).

*Felt Arousal Scale* (*FAS*): Activation/arousal was recorded with the Felt Arousal Scale of the Telic State Measure (45). This is a single-item self-report measure used extensively in exercise research (44, 46, 47). This 6-point scale ranges from 1 (*low arousal*) to 6 (*high arousal*). Correlations of the FAS with the SAM arousal scale range from .45 to .70. Correlations with the arousal scale of the AG range from .47 to .65.

*Health behaviors over the last two + hours:* Recent eating, sleeping, and exercise behaviors were assessed with three similar multiple-choice questions, with options indicating actions done 0–30 min ago, 30–60 min ago, 1–2 h ago, and 2 + hours ago. Eating had the additional option of “I am eating right now” and exercise had additional options of “I am exercising right now” and “I haven’t exercised yet today”.

*Health behavior intentions for the next two + hours*: Future eating, sleeping, and exercise intentions were assessed with 3 multiple choice questions, asking when participants next planned to sleep, eat, and exercise. Options included “in 0–30 min”, “in 30–60 min”, “in 1–2 hours” and “in 2 + hours”. For exercise, there was an additional option of “I am not going to exercise for the rest of the day”.

*Body position*: Body position was recorded with a multiple-choice list of lying down, sitting, standing (while leaning on something), standing (upright, not leaning), walking, exercising (other than walking), and other (please specify).

*Bathroom Urge:* Additionally, participants recorded how much of an urge they felt to use the restroom at the end of the survey on a Likert scale of 1–5. The urge to urinate is highly related to desires to move, which can confound data. It also was used as an indicator of any problems with the other data.

#### Data analysis

To provide evidence bearing on the research questions outlined above using longitudinal data, we utilized hierarchical linear modeling (HLM, multilevel modeling) with observations (Level 1) nested within participants (Level 2). This resulted in 1,031 observations nested within the 21 participants with complete and valid data. We followed the recommendations of Raudenbush and Bryk (48). Thus, we first computed an intercepts only model to ensure that subsequent models provided a better fit to the data. Concerning CRAVE move scores, the intercepts only model showed that CRAVE scores significantly differed [*b *= 17.71, *p *< .001, CI_95%_ (15.68, 19.73)] in the absence of any predictors. Between participant differences accounted for 12% of the observed variance in CRAVE move scores (*ICC* = .12). Similarly, CRAVE rest scores significantly differed in the intercepts only model [*b *= 21.43, *p *< .001, CI_95%_ (18.01, 24.85)] and showed more between subject variability (22%, *ICC* = .22) than observed for CRAVE move scores.

For each model containing a Level 1 predictor, we evaluated both random intercepts and random coefficients models retaining the model that provided the best fit to the data. To ensure concise reporting all model information is presented in relevant tables and text simply describes the nature of the observed relationships. All analyses were computed in *R* (version 4.1.2 [2021-11-01] (49) using the LME4 package (50), which incorporates Satterthwaite's degrees of freedom method (51). When using CRAVE scores to predict binary behavioral intentions, we used the general linear model (glmer) and specified the binomial family of distributions, which is appropriate when conducting binary logistic multilevel models on binary outcome data. Of note, odds ratio values less than one indicate that an increase in the *X* variable results in a decrease in the *Y* variable, and for odds ratios greater than one an increase in *X* corresponds with an increase in *Y*. The odds ratios also indicate the likelihood of an increase in *Y* given a one unit increase in continuous *X*. For example, CRAVE move scores significantly predict whether one intends to not stand (0) or stand (1). We observed an odds ratio of 1.05. This indicates that for every one unit increase in CRAVE move scores the likelihood of intending to stand were 1.05 times higher compared to not intending to stand.

Longitudinal data was also analyzed with Cosinor analysis to determine if participants had a circadian waveform. This analysis assumes either a normal or gamma distribution for outcomes. Cosinor parameters include mesor, acrophase, amplitude, nadir, and a test for rhythmicity. Such an analysis has been used for diurnal variations in heart rate and sleep (52), salt sensitivity in hypertension (53), peak expiratory flow in COPD (54), blood cardiac troponin T concentration (55) and others. Each participant's data was analyzed separately per the method developed by Doyle et al. (52). If either beta value was significant (*p* < .05), it was considered a circadian curve. Data was visually inspected with predicted curves for verification.

## Results

Means, standard deviations, and correlations between outcome variables for baseline and the final day are presented in Table 1. Collapsed across all measurement periods, the mean (SD) for Move-Now was 17.74 (SD* *= 12.97) and for Rest-NOW was 21.35 (SD* *= 16.25).

**Table 1:** Correlation matrices demonstrating associations between CRAVE factors with pleasure/displeasure and arousal/activation, both as measured “right now” (A) and “over the past week” (B).

(A) “Right now” (RN)	CRAVE-Move-RN @baseline	CRAVE-Rest-RN @baseline	Pleasure/displeasure-RN @baseline	Arousal-RN @baseline	CRAVE-Move-RN @last day	CRAVE-Rest-RN @last day	Pleasure/displeasure-RN @last day	Arousal-RN @last day
CRAVE-Move-RN @baseline	1	−0.68**	0.18	0.54*	−0.01	−0.02	−0.16	−0.17
CRAVE-Rest-RN @baseline	** **	1	−0.38	−0.29	−0.10	0.54*	−0.16	0.02
Pleasure/displeasure-RN @baseline	** **		1	−0.13	0.14	−0.44*	0.51*	0.08
Arousal-RN @baseline	** **			1	0.25	0.05	−0.29	0.27
CRAVE-Move-RN @last day	** **				1	−0.55**	0.19	0.78**
CRAVE-Rest-RN @last day	** **					1	−0.58**	−0.35
Pleasure/displeasure-RN @last day	** **						1	0.32
Arousal-RN @last day								1
Mean and standard deviation	21.48 ± 11.33	20.95 ± 15.33	2.14 ± 1.85	2.29 ± 1.01	21.19 ± 11.44	15.43 ± 13.11	2.24 ± 2.14	2.29 ± 1.35
(B) “Past week” (PW)	CRAVE-Move-PW @baseline	CRAVE-Rest-PW @baseline	Pleasure/displeasure-PW @baseline	Arousal-PW @baseline	CRAVE-Move-PW @last day	CRAVE-Rest-PW @last day	Pleasure/displeasure-PW @last day	Arousal-PW @last day
CRAVE-Move-PW @baseline	1	−0.70**	0.53*	0.49*	0.55**	−0.65**	0.31	0.63**
CRAVE-Rest-PW @baseline	** **	1	−0.49*	−0.17	−0.35	0.87**	−0.48*	−0.42
Pleasure/displeasure-PW @baseline	** **		1	0.01	0.26	−0.54*	0.45*	0.32
Arousal-PW @baseline	** **			1	0.48*	−0.07	0.30	0.71**
CRAVE-Move-PW @last day	** **				1	−0.48*	0.33	0.52*
CRAVE-Rest-PW @last day	** **					1	−0.52*	−0.33
Pleasure/displeasure-PW @last day	** **						1	0.29
Arousal-PW @last day								1
Mean and standard deviation	30.10 ± 8.68	17.14 ± 13.87	1.81 ± 1.91	3.33 ± 1.20	28.19 ± 9.39	16.00 ± 11.61	2.33 ± 1.77	2.71 ± 1.0

* *p* < .05.

** *p* < .01.

### Do movement and rest wants/desires vary across the day? How do they vary?

#### Hierarchical linear modelling

**Figure 2:**
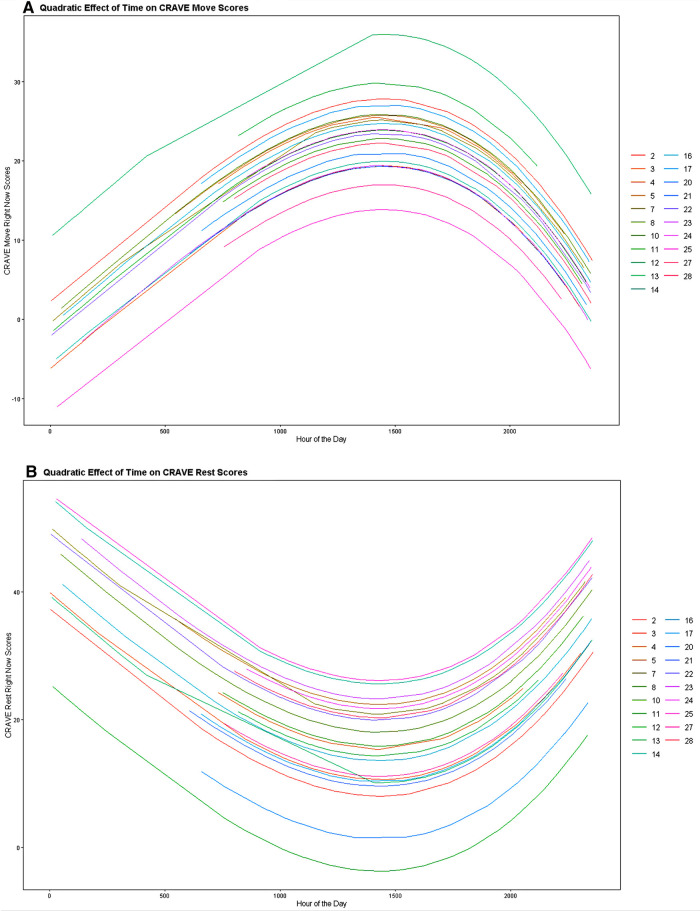
Linear and quadratic associations of time with motivation states to move (**A**) and rest (**B**). Note that on initial inspection, these figures seem to be perfect mirrored images—which suggests that Move and Rest desire are measuring either end of a singular construct. However, looking closely at the colors reveals that the rank order shifts across participants, and there is a smaller correlation between Move and Rest than at first glance.

**Figure 3:**
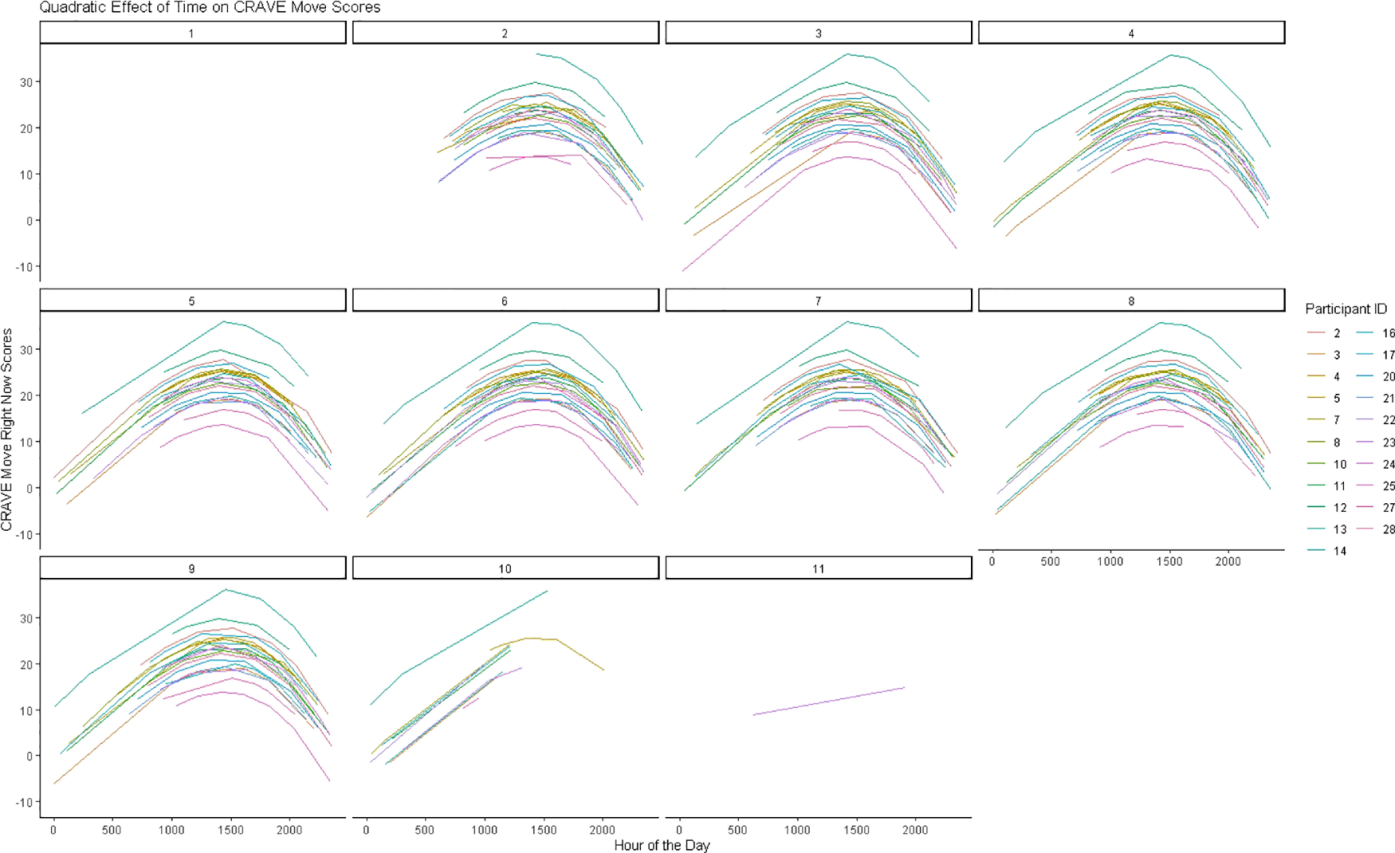
Predicted changes in move motivation states over 8 days.

To determine the influence of time on CRAVE move and rest scores we regressed the linear, cubic, and quadratic trends of time on CRAVE scores while allowing intercepts to vary across participants. When considering CRAVE move scores, we observed significant linear [*b *= .024, *p *< .001, CI_95%_ (.012,.035)] and quadratic time trends (*b *= −0.0000000054, *p *< .001, CI_95%_ [−0.0000000081, −0.0000000027]; the cubic time trend was non-significant (*p* =.49). CRAVE move scores increased from 0000 h until 1500 h and decreased from 1500 h to 0000 h (Figure 2A). As shown in Figure 3, this pattern was consistent when both collapsing across days and examining individual daily recordings.

**Figure 4:**
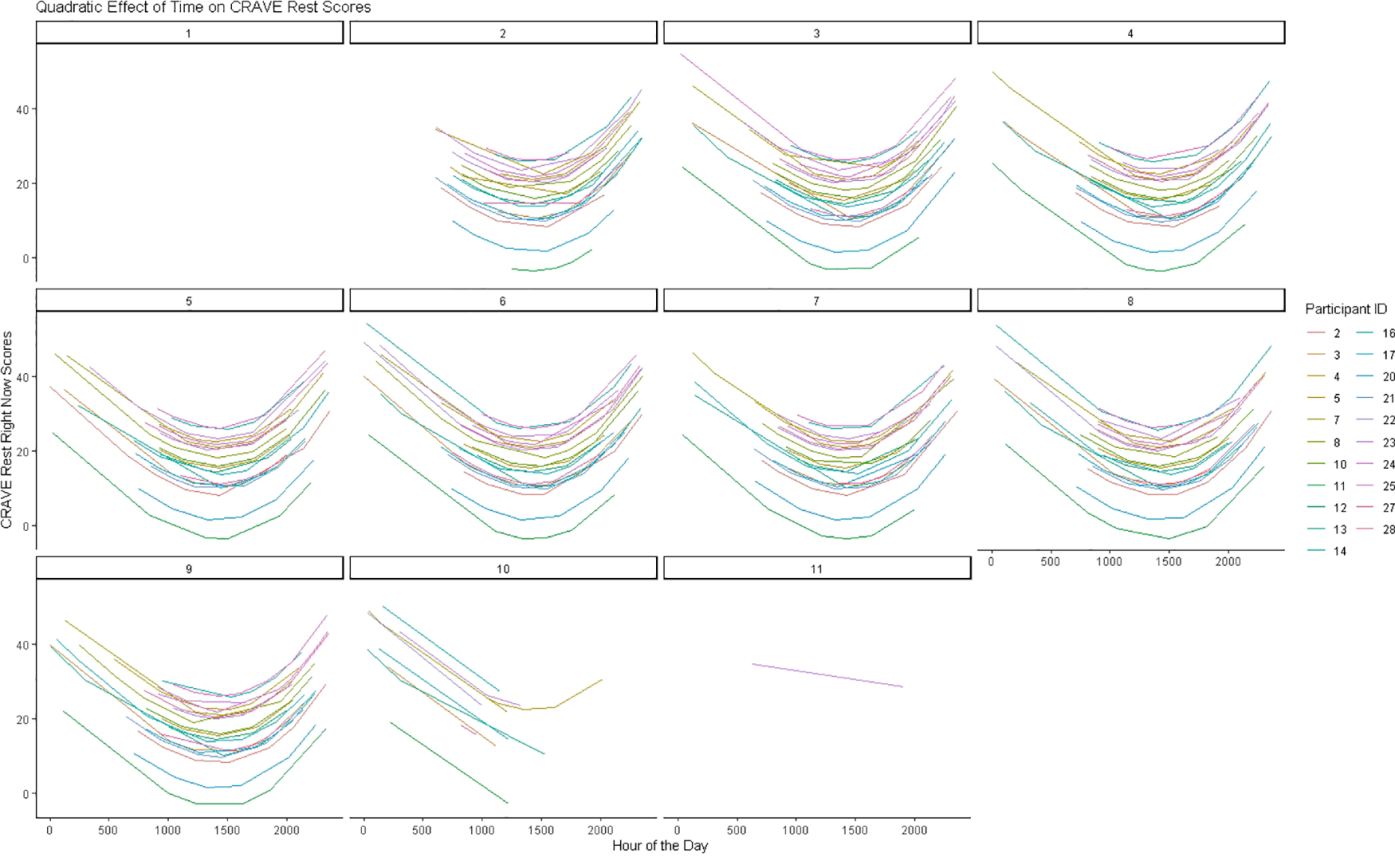
Predicted changes in rest motivation states over 8 days.

Time also showed significant linear (*b *= −.030, *p *< .001, CI_95%_ [−0.044, −0.017] and quadratic trends [*b *= 0.0000000051, *p *= .002, CI_95%_ (0.000000018, 0.0000000083)] on CRAVE rest scores. Examination of Figure 2B indicates that CRAVE rest scores decreased from 0000 h until 1500 h at which time they increased from 1500 h until 0000 h. This pattern also occurred both when collapsing across days and examining daily variation (see Figure 4).

#### Cosinor analysis

**Figure 5:**
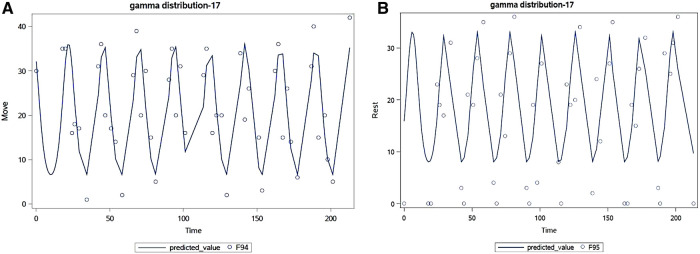
Example cosinor analysis for participant 17 for move (**A**) and rest (**B**) over 200 h.

Cosinor analysis found that 81% of participants had a circadian curve for Move and 62% had one for Rest. See Figures 5A,B for examples of these analyses for participant 17. Thus, both CRAVE Move and Rest scores appear to vary across the day and exhibit a quadratic pattern where scores increase or decrease early in the day and then begin to reverse during mid afternoon.

### Is there an association with pleasure?

#### Move

At Days 1 and 8, Move-NOW correlations with pleasure (assessed “now”) were small (Day 1: *r* = .18, Day 8: *r* = .19).

The random coefficients model best fit the data when using pleasure to predict CRAVE move scores (*X*^2^[2] = 62.29, *p *< .001) with approximately 28% of the variance due to participant clustering (*ICC *= .28). For each unit increase in felt pleasure right now, CRAVE move scores increased 3.38 units [*b *= 3.38, *p *< .001, CI_95%_ (2.40, 4.37)]. Importantly, this result was similar even when controlling for linear and quadratic time trends [*b *= 2.96, *p *< .001, CI_95%_ (2.17, 3.76)].

#### Rest

At Days 1 and 8, Rest-NOW correlations with pleasure were small to moderate (Day 1: *r* = −.38, Day 8: *r* = −.58).

Similarly, the random coefficients model presented the best fit to the data when predicting CRAVE rest scores from felt pleasure right now [*X*^2^(2) = 49.74, *p *< .001] with between subject clustering accounting for approximately 27% of the variance (*ICC *= .27). For each unit increase in felt pleasure right now, CRAVE rest scores tended to decrease by 3.91 units [*b *= −3.91, *p *< .001, CI_95%_ (−5.02, −2.81)]. This finding also held even when controlling for the linear and quadratic time trends [*b *= −3.41, *p *< .001, CI_95%_ (−4.31, −2.51)]. Therefore, pleasure felt in the current moment explains unique variance beyond that explained by the time trends in CRAVE move and rest scores.

These finding suggest there is a relationship between pleasure, and both CRAVE Move and Rest scores. Specifically, as felt pleasure increases Move scores increase, and Rest scores decrease.

### Is there an association with arousal?

#### Move

At Days 1 and 8, Move-NOW correlations with arousal/activation (assessed “now”) were moderate (Day 1: *r* = .54, Day 8: *r* = .78).

The random coefficients model exhibited the best fit to the data [*X*^2^(2) = 80.72, *p *< .001] with between-subject effects accounting for 28% of the variance in CRAVE move scores (*ICC *= .28). In this model each one unit increase in felt arousal right now predicted a 6.39 unit increase in CRAVE move scores [*b *= 6.39, *p *< .001, CI_95%_ (5.03, 7.74)]; these results remained consistent [*b *= 5.47, *p *< .001, CI_95%_ (4.26, 6.67)] even when statistically accounting for any potential influence of linear or quadratic time.

#### Rest

At Days 1 and 8, NOW correlations with arousal/active were small (Day 1: *r* = −.35, Day 8: *r* = −.35).

When examining CRAVE rest scores, results suggest that the random coefficients model best fit the data [*X*^2^(2) = 69.09, *p *< .001]. Between-subject clustering accounted for 39% of the variance in CRAVE rest scores (*ICC *= .39). For each one unit increase in felt arousal right now, CRAVE rest scores tended to decrease by 6.49 units [*b *= −6.4, *p *< .001, CI_95%_ (−8.14, −4.84)]. Importantly, these results held and were similar in magnitude [*b *= −5.35, *p *< .001, CI_95%_ (−6.83, −3.88)] even when statistically controlling for both the linear and quadratic effects of time. Together these results suggest that despite an observed time variation in CRAVE move and rest scores, increased felt arousal uniquely increases CRAVE move and decreases CRAVE rest scores.

Thus, arousal does associate with both CRAVE Move and Rest scores in a similar pattern as observed for pleasure. As arousal increased Move scores increased and Rest scores decreased.

### Do pleasure and arousal interact on CRAVE scores?

To explore whether pleasure and arousal present additive or multiplicative effects on CRAVE scores we next examined more complex models where pleasure, arousal, and their interaction term predicted CRAVE scores. Concerning CRAVE move scores, the random coefficients model provided the best fit to the data [*X*^2^(5) = 112.58, *p *< .001]. The results suggested additive effects such that both self-reported pleasure [*b *= 1.14, *p *= .011, CI_95%_ (0.26, 2.02)] and arousal [*b *= 5.46, *p *< .001, CI_95%_ (3.97, 6.95)] predicted increased CRAVE move scores. The pleasure and arousal interaction failed to achieve significance in this model [*b *= .13, *p *= .26, CI_95%_ (−0.10, 0.36)].

When examining CRAVE rest scores, the random coefficients model also represented the best fit to the data [*X*^2^(5) = 73.33, *p *< .001]. In this model, increased pleasure [*b *= −2.14, *p *< .001, CI_95%_ (−3.21, −1.07)] and arousal [*b *= −5.90, *p *< .001, CI_95%_ (−7.69, −4.12)] predicted decreased CRAVE rest scores. The interaction term also failed to achieve traditional significance levels when examining the pleasure and arousal interaction in this model [*b *= .17, *p *= .26, CI_95%_ (−0.13, 0.47)]. These results suggest that both perceived pleasure and arousal uniquely (additively) contribute variance when predicting CRAVE move and rest scores.

Our results do not suggest a pleasure by arousal interaction on CRAVE scores. Rather these results indicate that both pleasure and arousal additively influence CRAVE.

### Do previous behaviors impact wants/desires for movement and rest?

To determine whether previous behaviors predicted CRAVE move or rest scores we examined several outcomes of relevance. For each of the analyses reported in the following, the predictor variable was coded 0 (an absence of that behavior) or 1 (engaging in that behavior). In-text discussion is centered on significant findings; however, full results for all predictor variables are available in Table 2.

**Table 2:** Previous behaviors predicting CRAVE move and rest scores.

Predictor Variables	CRAVE Move Scores			CRAVE Rest Scores		
	*b*	CI_95%_	*p*	*b*	CI_95%_	*p*
Currently Eating	*2*.*63*	*−0.01, 5.27*	.*05*	**−4**.**00**	**−7.12, −0.89**	.**01**
Ate 0 to 30 Minutes Ago	0.88	−1.24, 2.99	.42	−1.57	−4.06, 0.92	.22
Ate 30 to 60 Minutes Ago	2.02	−0.59, 4.63	.13	−1.53	−6.40, 1.55	.33
Ate 1 to 2 Hours Ago	**2**.**63**	**0.62, 4.65**	.**01**	−2.04	−4.42, 0.35	.09
Ate Over 2 Hours Ago	**−3**.**68**	**−6.31, −1.04**	.**01**	**3**.**92**	**0.74, 7.10**	.**02**
Did Not Exercise Today	**4**.**80**	**1.23, 8.39**	.**01**	**−6**.**44**	**−10.31, −2.57**	.**001**
Exercising Now	**16**.**94**	**11.70, 22.19**	**<**.**001**	**−13**.**32**	**−19.58, −7.06**	**<**.**001**
Exercised 0 to 30 Minutes Ago	0.71	−2.74, 4.17	.69	−0.87	−4.95, 3.20	.67
Exercised 30 to 60 Minutes Ago	*3*.*39*	*−0.05, 6.82*	.*05*	−2.88	−6.93, 1.18	.16
Exercised 1 to 2 Hours Ago	−0.16	−3.30, 2.99	.92	1.09	−2.61, 4.80	.56
Exercised Over 2 Hours Ago	**−8**.**51**	**−11.01, −6.01**	**<**.**001**	**9**.**83**	**6.99, 12.66**	**<**.**001**
Slept 0 to 30 Minutes Ago	**−6**.**06**	**−9.74, −2.38**	.**001**	*4*.*72*	*−0.13, 9.57*	.*056*
Slept 30 to 60 Minutes Ago	−1.67	−6.62, 3.28	.51	1.32	−4.84, 7.47	.68
Slept 1 to 2 Hours Ago	**4**.**60**	**1.02, 8.18**	.**01**	−3.59	−7.82, 0.65	.10
Slept Over 2 Hours Ago	**2**.**77**	**0.98, 4.57**	.**002**	**−2**.**68**	**−4.80, −0.56**	.**01**

*Note.* **Bold **= significant at *p *< .05; *underlined italics* = *p* between .05 and .06.

Participants who reported eating 1 to 2 h before the survey, not exercising on the survey day, exercising while completing the survey, sleeping 1 to 2 h before the survey, and sleeping over two hours before the survey also reported higher CRAVE move scores. Yet participants who ate over two hours before the survey, exercised over two hours before the survey, and slept zero to 30 min before the survey tended to report lower CRAVE move scores. As shown in Table 2, all other variables failed to contribute significant variance to predicting CRAVE scores.

Concerning CRAVE rest scores, eating during the survey, not exercising on the day of the survey, exercising during the survey, and sleeping over two hours before the survey each resulted in lower CRAVE rest scores. Eating over two hours before the survey and exercising over two hours before the survey resulted in increased CRAVE rest scores. All other predictors failed to explain unique variance in CRAVE rest scores. Overall, these findings suggest some past behaviors do influence both Move and Rest scores.

### Do wants/desires for movement and rest impact future behavioral intentions? (multilevel logistic regression analyses)

As noted in the Data Analysis section, to determine whether CRAVE move and rest scores influence behavioral intentions, we entered both move and rest scores as predictors of the various behavioral intentions in binary logistic multilevel models (0 = absence of the behavior; 1 = presence of the behavior). We observed that for each unit increase in CRAVE move scores the likelihood of currently being in a standing position, currently walking, engaging in other exercise, exercising now, exercising 0 to 30 min later, exercising 30 to 60 min later, and sleeping over 2 h later were higher. Alternatively, for each unit increase in CRAVE move scores the likelihood of intending to sit during the survey, sleep 0 to 30 min later, and sleep one to two hours later was lower. We also observed that for each unit increase in CRAVE rest scores, the likelihood of lying down during the survey and to sleep zero to 30 min later were higher. Yet for each unit decrease in CRAVE rest scores the likelihood of intending to exercise one to two hours later, exercise over two hours later, and sleep over two hours later were lower. Overall, these results suggest that CRAVE scores likely can predict future behavioral intentions. See Table 3.

**Table 3:** CRAVE move and rest scores predicting body position, exercise and eating at the time of the surveys and future intentions to eat, exercise and sleep 2 + hours into the future.

Dependent Variables	CRAVE Move				CRAVE Rest			
	Log-0dds	Odds ratio	Predicted %	*p*	Log-Odds	Odds ratio	Predicted %	*p*
Lying Down	−0.041	–	–	0.07	**0**.**072**	**1.07**	**0.52**	**<**.**001**
Sitting	−**0**.**030**	**0.97**	**0.49**	**0**.**01**	−0.017	–	–	0.10
Leaning on Something	0.006	–	–	0.81	−0.030	–	–	0.16
Standing	**0**.**052**	**1.05**	**0.51**	**0**.**005**	−0.016	–	–	0.31
Walking	**0**.**118**	**1.13**	**0.53**	**<**.**001**	0.004	–	–	0.91
Other Exercise	**0**.**131**	**1.14**	**0.53**	**0**.**036**	−0.061	–	–	0.49
Currently Eating	0.001	–	–	0.97	−0.016	–	–	0.27
Eating 0 to 30 Minutes Later	−0.025	–	–	0.22	−0.031	–	–	0.07
Eating 30 to 60 Minutes Later	0.013	–	–	.40	0.015	–	–	0.25
Eating 1 to 2 Hours Later	0.017	–	–	0.19	−0.016	–	–	0.16
Eating Over 2 Hours Later	−0.012	–	–	0.26	0.009	–	–	0.31
Exercising Now	**0**.**177**	**1.19**	**0.54**	**<**.**001**	0.049	–	–	0.21
Exercising 0 to 30 Minutes Later	**0**.**098**	**1.10**	**0.53**	**<**.**001**	0.004	–	–	0.86
Exercising 30 to 60 Minutes Later	**0**.**077**	**1.08**	**0.52**	**0**.**005**	0.004	–	–	0.86
Exercising 1 to 2 Hours Later	0.014	–	–	0.45	**−0**.**039**	**0.96**	**0.49**	**0**.**024**
Exercising Over 2 Hours Later	−0.010	–	–	0.44	**−0**.**033**	**0.97**	**0.49**	**0**.**004**
Sleeping 0 to 30 Minutes Later	**−0**.**0279**	**0.76**	**0.43**	**<**.**001**	**0**.**08**	**1.08**	**0.52**	**0**.**005**
Sleeping 30 to 60 Minutes Later	−0.058	–	–	0.28	0.074	–	–	0.07
Sleeping 1 to 2 Hours Later	**−0**.**059**	**0.94**	**0.49**	**0**.**04**	0.018	–	–	0.39
Sleeping Over 2 Hours Later	**0**.**150**	**1.16**	**0.54**	**<**.**001**	**−0**.**069**	**0.93**	**0.48**	**<**.**001**

*Note*. **Bold** = significant at *p* < .05.

## Discussion

The current data provide novel insights into the dynamics of motivation states for physical activity and rest—they vary diurnally, are influenced by recent behaviors (e.g., exercise, eating and sleep), and predict future intentions to be active. Importantly, this is the first study to demonstrate that the motivation to move and be sedentary in humans varies like a biorhythm. Using both hierarchical linear modeling (HLM) and Cosinor analysis, we found that desires/urges to move and rest followed a circadian pattern, with a peak around 1500 h for Move and a similar nadir for Rest. Also, for the first time, recent eating and sleeping were found to be associated with current motivation states to move and rest. Exercise was particularly related with these desires. In logistic regression models, motivation states to move and rest predicted current exercise and body position (e.g., standing, walking), which is what one would expect, thus providing additional validation of the CRAVE scale (10). More importantly, motivation states predicted intentions to exercise and sleep in the near term (i.e., 0–2 h). Of note, this is the first study to make this conclusion in a healthy population. Lastly, feeling states were associated with desire to move and rest, with arousal/activation having nearly twice the influence as pleasure/displeasure. These data compliment and augment what we have found from 7 previous studies investigating motivation states—specifically, people have transient desires to move and rest that are influenced by previous behaviors (9–12, 14).

The major finding from this investigation was that motivation states vary in a manner that is like a circadian curve. Cosinor analysis found that 81% of individuals had a circadian curve for Move and 62% for Rest. Why Rest was lower is difficult to explain but may be due to the dysregulated sleeping patterns commonly found today (56). Many biological variables are under circadian control, including cortisol (with a peak 30 min after awakening), blood pressure, sex hormones (e.g., testosterone peaks in the afternoon), growth hormone (e.g., covarying with REM sleep), body temperature, and other biomarkers (57–60). Positive and negative affect (61), as well as sensations of energy, fatigue, and pain, have also been found to vary in a circadian manner, for some individuals (59). Some researchers have emphasized that changes in motivation are due to random factors (20) or may be more functional, such as in deprivation and satiation models (62), or toggle between states of exploration (i.e., leisure) and exploitation (i.e., labor), as in the Elaborated Process of self-regulation (22). Our data could be complementary with these models, but they largely suggest that motivation states are highly influenced by diurnal factors. See Stults-Kolehmainen et al. (12) for greater discussion.

Recent (i.e., 0–2 h) exercise and sleeping behaviors were associated with motivation states to move and rest in a very complicated set of associations. As one might expect, during exercise, desire to move was higher and desire to rest was lower, both by more than one standard deviation. Two or more hours after exercise, the opposite occurred (>½ standard deviation for both). Between these times, there was no association. This pattern may be due to differences in the transient feelings that follow exercise. Some have an exercise afterglow with a bout of exercise, while others are fatigued (63); numerous interpersonal and exercise-related factors likely have an influence on motivation (64). For sleep, it was clear that recent awakening was associated with less desire to move and more to rest, which conforms to what is known about sleep inertia (65). The reverse was true two hours after awakening. These data are concordant with previous investigations that periods of heightened movement and rest result in changes in desire to move (10, 12). In these studies, however, we found that maximal exercise had an immediate and large impact on motivation states (i.e., Move decreases, Rest increases), and periods of prolonged sitting resulted in small to moderate increases in the desire to move. Further studies should elucidate how different exercise modes and intensities may modulate these changes.

Eating was associated with the desire to move and rest—also in a complicated fashion. First, Move and Rest motivation states were associated with current eating behavior– which is slightly counter-intuitive as it seems like one would not want to be moving during eating. There are various explanations for this observation. First, in the modern era of multi-tasking, feeding times are frequently utilized to watch media (66), complete various chores and responsibilities, and prepare for upcoming important tasks (67). Second, there may simply be greater energy availability from ingesting nutrients, spurring motivation. Nevertheless, digestion is a process that takes time, which is a counterargument. Third, some data demonstrate that demonstrate that even a simple rinse of carbohydrate in the mouth is sufficient to spur effort for movement, perhaps by activating motivation centers of the brain (68). Fourth, the simple movements of lifting the hand to the mouth during eating might be construed as relevant by some participants. Indeed, participants in former studies have indicated this (12). Interestingly, having eaten 1–2 h ago was associated with greater desire to move, but 2 + hours was associated with less. Again, this might make sense from the standpoint of digestion and blood glucose kinetics and autonomic responses during digestion (69). There might be an optimal period of energy availability, which would promote greater desire to move. It also seems to align with advice with various sports nutrition experts that meals should be timed appropriately before a workout (70) depending upon the individual need of nutrients and the exercise demands. Further research is warranted on this issue given the complexity of these factors and their potential interactions.

Current body position and future health behavior intentions (for exercise and sleep) were predicted by motivation states to move and rest. There was a clear pattern of influence of motivation states to move on position of the body. Sitting was associated with lower Move scores. Standing, walking, and exercise were associated with higher Move (in that order). Lying down, on the other hand, was associated with greater Rest. Importantly, current exercise and future intentions for exercise up to 1 h was predicted by Move, with exercising at the time of the survey associated with the strongest desire to move, as one might expect. Intentions for behavior greater than 1 h in advance were not associated with Move and Rest, with the exception of plans to sleep > 2 h, which were associated with greater Move and less Rest desire. Log odds in the logistic regression indicate that a one-point increase in motivation to move was associated with a 1.10 times (53%) greater likelihood of intending to exercise in the next 30 min and a 1.08 times (52%) greater likelihood of intending to move in the next 30–60 min. Finally, neither desires to move nor rest predicted future eating intentions—a behavior most closely linked to the desire for food (67).

The current study provides additional evidence of the validity of the concept of ACMS for movement and rest, and for the WANT model of motivated behavior for physical activity. These postulates were supported: (A) that desires are separate, (B) they are highly transient, (C) they change based on previous behaviors, (D) they work loosely and asynchronously, and (E) they differ from emotions and psychosomatic sensations. Each are explicated below.

A) As with previous studies (10–12), there were moderate correlations (−.68, −.55) between desires to move and rest/be sedentary.
B) There were significant linear and quadratic effects of time on Move and Rest scores, indicating steady change across the day.
C) Eating, sleeping and exercise behaviors all influenced subsequent CRAVE assessments.
D) The differential influences of motivation states for movement and rest on health behaviors, particularly exercise and sleep, provide some support for the tenet that ACMS work loosely and asynchronously. While we found no evidence that they were totally concordant (e.g., desires to move and rest changing the same direction), there was evidence that body position and exercise behavior had varying influences on the desire to move or rest.
E) Motivation states were associated with both pleasure/displeasure and arousal (activation). Furthermore, activation had nearly twice the influence of feeling states. Our previous studies have found that motivation states are also related to perceived energy and fatigue (10). These data appear to support the idea that motivation states have a strong affective component, which may be felt as tension, and have been called affectively-charged motivation states (ACMS) (30). Perhaps it's worth noting that while a substantial portion of the variance was explained by affect and arousal, there was substantial variance in CRAVE move and rest scores at Level 2, the person level of analysis. This variance likely reflects the influence of individual differences (e.g., personality, trait move/rest preferences) that may modify the reported relationships—an avenue for future research (71, 72).

Motivation states likely derive from a variety of inputs, including: (1) a basic drive to move (23, 73), (2) necessity of movement to accomplish tasks (simple instrumental value), (3) reflection (26, 74), and (4) reward (75). These relationships may further differ based on individual traits.

### Study limitations

Despite the novelty and importance of these data, there were some limitations. First, we did not screen for movement or sleep problems or diminished or excessive urges for movement and sleep, the so-called movement urge dysfunction disorders (MUDD) (14, 15). Furthermore, we did not assess movement and sedentarism with objective measures of exercise, physical activity, sleep, et cetera, nor did we assess for exogenous sources that may influence motivation states, like caffeine (76, 77), medication use (78), or environmental factors known to strongly affect motivation, like music (31). Unfortunately, little is known about participants' background (e.g., employment status, income, occupation, normal work hours, and overall health status), all of which may be relevant. For instance, nurses working third shift have altered physical activity patterns (79), have a larger body mass index, and frequently suffer from fatigue and declining health (80).

Additionally, the sample size at level two of our statistical models was *n* = 21. The current literature has yet to delineate clear guidelines regarding the optimal number of clusters required for a multilevel model to be considered adequately powered; suggestions range from as little as 10 clusters to as many as 50 or more clusters depending on model complexity, design, the number of observations within each cluster, and other considerations (81, 82). We had 1,031 observations at level one, and we did not test any level two predictors in our models. Given that person-level (level two) variance explained approximately 12% of differences in CRAVE-Move and 22% of differences in CRAVE-Rest scores in the absence of predictors, our models simply controlled for those person-level differences (allowed individual slopes to vary) to focus on the relationships among the level one predictor variables and CRAVE-Move and Rest scores. Future research could build on this work by examining person-level factors (i.e., individual differences, such as personality, need for stimulation, et cetera) that influence baseline CRAVE-Move and Rest score differences.

Still the current approach is valid given that modeling level two variance (accounting for unmeasured individual differences) is particularly important, especially when the numbers of clusters is small (83). Our models also used restricted maximum likelihood (REML) for estimation– a method shown to perform well even with 10 or fewer clusters (83–85). Thus, our statistical approach is consistent with recent suggestions in the literature for analyzing multilevel data with small level two sample sizes. Similar work examining the influence of variables nested within individuals across time appears in the literature and reports similar level two sample sizes as collected in this work (e.g., (83, 86, 87). Still, as with any research, future work collecting larger samples is necessary to further confirm these results and extend generalizability to larger and more diverse populations. We utilized two sophisticated analytic techniques, but each comes with their own limits, and there does not appear to be a perfect technique for the analysis of circadian data. For instance, the cosinor approach may be too restrictive for some individuals, as it assumes that the circadian pattern is always a cosine shape (19).

### Future research

Future research possibilities have been extensively discussed in our recent manuscripts (9, 10, 12, 14), but regarding these data, several studies are suggested as follow ups.

1. Examine whether motivation states predict actual physical activity and rest/sedentary behaviors with both experimental procedures in the laboratory and in natural settings.
2. Understand why specific behaviors (e.g., eating, sleeping) seem to hold influence relative to others on motivation states.
3. Determine the frequency of mismatch between desires to move and the ability to move, given modern environments that constrain movement.
4. Compare the influence of avoidance motivation (e.g., aversions/diswants) on activity and rest, as depicted by the WANT model, in relation to approach motivation.
5. Examine motivation and affective states during task (i.e., during exercise), which is now possible because single-item versions of the CRAVE subscales were recently developed in both English and Portuguese (11).
6. Understand how motivation states fluctuate during recovery from exercise, because the experience of affect during this period predicts future exercise behavior (88).
7. Calculate variations over other time frames, such as weekly, seasonally or annually (89).
8. Conduct experiments to determine the relationship of CRAVE (motivation to move and rest) with biomarkers that also vary in a circadian pattern, such as sex hormones and cortisol.
9. Understand whether various kinds of disease and disorders are associated with disrupted circadian rhythmicity of motivation states, such as Alzheimer's (90).
10. Differentiate motivation states for various chronotypes, including larks (morningness chronotype) vs. night owls (evening chronotype), or alternatively, roadrunners (active in the afternoon), penguins (low overall activity), hummingbirds (high overall activity) and other proposed chronotypes (19, 61).
11. Conduct just-in-time adaptive interventions (JITAI) (91) to maximize opportunities when people experience “CRAVE moments” (moments when the desire to move is high), and perhaps intervene to promote desires when they are low.
12. Given the initial nature of this work, we did not conduct cross-lagged analyses of the data; however, future work should consider how CRAVE scores on one day influence important outcomes on following days.

## Application

These data likely have real world application for the promotion of physical activity, exercise training and even workplace productivity. For those wishing to maximize the effectiveness of exercise, it may make sense to align training sessions with a time frame when motivation for movement is naturally high instead of attempting to generate motivation at times when it is lacking. For an average person, peak motivation to move is around 15:00 h, so it may make sense to exercise on the incline before the peak (∼14:00–15:00 h) so peak motivation coincides with the end of exercise. Working out >2 h after awakening may be sufficient, at least not close to bedtime. It also appears that motivation is higher in a window of 1–2 h after eating.

Desire to move is associated with higher levels of pleasure/lower levels of displeasure. More pertinently, higher levels of arousal/activation are associated with the desire to move to an even greater degree. This suggests that one strategy to improve motivation to move is by promoting incidental affect, hedonic tone and, perhaps, by energizing action. Psychological stress and poor mood are well-established barriers for physically active behaviors (12, 92–95). When faced with these situations, it would be helpful to connect individuals who are suffering with resources to help them cope, or to lower barriers elsewhere for initiating physical activity. It seems likely that many individuals are most motivated to move during the workday, and in the evening time motivation to move is diminishing—the time when most people attempt to go to the gym (96). Given this, it may make sense to promote movement in the workplace. Such a strategy may also improve workplace productivity (97). An intervention may be as simple as encouraging workers to stand up and move, which we demonstrated was associated with greater desires to be physically active (but not lesser desire to rest). While this is not a causal relationship, one might imagine that the simple act of standing up might promote desires to move, which can be taken advantage of later down the line. Qualitative data supports the ideas of inertia and momentum as strong forces impacting the desire to move (12). Finally, it is important to consider aspects of the exercise regimen, such as modality and intensity, which positively influence motivation for exercise by eliciting a positive affective response (98).

## Conclusion

This is the first study to investigate the natural variation of motivation for movement and sedentary behaviors across the day, finding that desires to move and rest resembled a biorhythm. Individuals wanted to move the most around 3:00 in the afternoon, approximately the same time their desire to rest was at its lowest. As with our former investigations, recent behavior (over the last 2 + hours) appeared to alter motivation states. Specifically, in the case of recent exercise, we observed that motivation states to move decreased (and to rest increased) two hours after exercise, but there was no change immediately afterwards. Current body position and current exercise behavior was strongly predicted by desires to move and rest indicating that when people are actually moving or in a state of readiness to move, they want to move. To our knowledge, this is also the first study to investigate the role of motivation states on future exercise behavior. Importantly, motivation states to move and rest predicted intentions for exercise and sleep in the near term (0–60 min). While recent eating behaviors predicted desires to move and rest, motivation states did not predict future eating intentions. Finally, motivation states were associated with feeling states, particularly arousal/activation. Overall, these data provided support that motivation states may be affectively-charged, short-lived, impacted by recent behavior, and associated with intentions to behave, as predicted by the WANT model (9).

## Data Availability

The raw data supporting the conclusions of this article are available at this location: https://www.researchgate.net/publication/369550926_CRAVE_circadian_official_dataset_Mar_27_2023.

## References

[1] Hyde ET Whitfield GP Omura JD Fulton JE Carlson SA. Trends in meeting the physical activity guidelines: muscle-strengthening alone and combined with aerobic activity, United States, 1998–2018. J Phys Act Health. (2021) 18(S1):S37–44. 10.1123/jpah.2021-0077 34465652 PMC11000248

[2] Ussery EN Whitfield GP Fulton JE Galuska DA Matthews CE Katzmarzyk PT Trends in self-reported sitting time by physical activity levels among US adults, NHANES 2007/2008–2017/2018. J Phys Act Health. (2021) 18(S1):S74–83. 10.1123/jpah.2021-0221 34465647 PMC8477754

[3] Williams DM Evans DR. Current emotion research in health behavior science. Emot Rev. (2014) 6(3):277–87. 10.1177/1754073914523052 PMC12975116 41815188

[4] Williams DM Rhodes RE Conner MT. Conceptualizing and intervening on affective determinants of health behaviour. Psychol Health. (2019) 34(11):1267–81. 10.1080/08870446.2019.1675659 31617431

[5] Brand R Ekkekakis P. Affective–reflective theory of physical inactivity and exercise. Ger J Exerc Sport Res. (2018) 48(1):48–58. 10.1007/s12662-017-0477-9

[6] Conroy DE Berry TR. Automatic affective evaluations of physical activity. Exerc Sport Sci Rev. (2017) 45(4):230–7. 10.1249/jes.0000000000000120 28704217

[7] Michie S van Stralen MM West R. The behaviour change wheel: a new method for characterising and designing behaviour change interventions. Implement Sci. (2011) 6(1):42. 10.1186/1748-5908-6-42 21513547 PMC3096582

[8] National Institute of Mental Health. Sensorimotor Dynamics (RDoc Constructs): National Institutes of Health (2022). Available at: https://www.nimh.nih.gov/research/research-funded-by-nimh/rdoc/constructs/sensorimotor-dynamics

[9] Stults-Kolehmainen MA Blacutt M Bartholomew JB Gilson TA Ash GI McKee PC Motivation states for physical activity and sedentary behavior: desire, urge, wanting, and craving. Front Psychol. (2020) 11(3076):2–13. 10.3389/fpsyg.2020.568390 33240154 PMC7677192

[10] Stults-Kolehmainen MA Blacutt M Fogelman N Gilson TA Stanforth PR Divin AL Measurement of motivation states for physical activity and sedentary behavior: development and validation of the CRAVE scale. Front Psychol. (2021) 12:4–19. 10.3389/fpsyg.2021.568286 33841225 PMC8027339

[11] Filgueiras A Stults-Kolehmainen MA Boullosa D Sinha R Bartholomew JB McKee P The CRAVE and ARGE scales for motivation states for physical activity and sedentarism: brazilian Portuguese translation and single-item versions. SportRxiv. (2022). 10.51224/SRXIV.224 PMC10495583 37705947

[12] Stults-Kolehmainen M Gilson T SantaBarbara N McKee P Sinha R Bartholomew J Qualitative and quantitative evidence of motivation states for physical activity, exercise and being sedentary from university student focus groups. SportRxiv. (2022). 10.51224/SRXIV.189 PMC10071436 37025458

[13] Antle MC Silver R. Circadian insights into motivated behavior. Behav Neurosci Motiv. (2015) 27:137–69. 10.1007/7854_2015_384 26419240

[14] Stults-Kolehmainen MA Blacutt M Bartholomew JB Boullosa D Janata P Koo BB Urges to move and other motivation states for physical activity in clinical and healthy populations: a scoping review protocol. Front Psychol. (2022) 13:5. 10.3389/fpsyg.2022.901272 35898999 PMC9311496

[15] Khan FH Ahlberg CD Chow CA Shah DR Koo BB. Iron, dopamine, genetics, and hormones in the pathophysiology of restless legs syndrome. J Neurol. (2017) 264(8):1634–41. 10.1007/s00415-017-8431-1 28236139

[16] Rosenberger ME Fulton JE Buman MP Troiano RP Grandner MA Buchner DM The 24-hour activity cycle: a new paradigm for physical activity. Med Sci Sports Exerc. (2019) 51(3):454–64. 10.1249/mss.0000000000001811 30339658 PMC6377291

[17] Di J Spira A Bai J Urbanek J Leroux A Wu M Joint and individual representation of domains of physical activity, sleep, and circadian rhythmicity. Stat Biosci. (2019) 11(2):371–402. 10.1007/s12561-019-09236-4 32440309 PMC7241438

[18] Xiao L Huang L Schrack JA Ferrucci L Zipunnikov V Crainiceanu CM. Quantifying the lifetime circadian rhythm of physical activity: a covariate-dependent functional approach. Biostatistics. (2014) 16(2):352–67. 10.1093/biostatistics/kxu045 25361695 PMC4804116

[19] McDonnell EI Zipunnikov V Schrack JA Goldsmith J Wrobel J. Registration of 24-hour accelerometric rest-activity profiles and its application to human chronotypes. Biol Rhythm Res. (2022) 53(8):1299–319. 10.1080/09291016.2021.1929673 35784395 PMC9246189

[20] Resnicow K Page SE. Embracing chaos and complexity: a quantum change for public health. Am J Public Health. (2008) 98(8):1382–9. 10.2105/AJPH.2007.129460 18556599 PMC2446457

[21] Resnicow K Vaughan R. A chaotic view of behavior change: a quantum leap for health promotion. Int J Behav Nutr Phys Act. (2006) 3(1):1–7. 10.1186/1479-5868-3-25 16968551 PMC1586207

[22] Inzlicht M Schmeichel BJ Macrae CN. Why self-control seems (but may not be) limited. Trends Cogn Sci (Regul Ed). (2014) 18(3):127–33. 10.1016/j.tics.2013.12.009 24439530

[23] Stults-Kolehmainen MA. Humans have a basic physical and psychological need to move the body: physical activity as a primary drive. SportRxiv. (2022). 10.51224/SRXIV.236 PMC10128862 37113126

[24] Casper RC Voderholzer U Naab S Schlegl S. Increased urge for movement, physical and mental restlessness, fundamental symptoms of restricting anorexia nervosa? Brain Behav. (2020) 10(3):e01556. 10.1002/brb3.1556 32017454 PMC7066368

[25] Iqbal N Lambert T Masand P. Akathisia: problem of history or concern of today. CNS Spectr. (2007) 12(9):1–13. 10.1017/s1092852900026201 17805218

[26] Williams DM Bohlen LC. Motivation for exercise: reflective desire versus hedonic dread. In: Anshel MH Labbé EE Petrie TA Petruzzello SJ Steinfeldt JA, editors. APA Handbook of sport and exercise psychology, volume 2: Exercise psychology. APA handbooks in psychology series. Washington, DC, US: American Psychological Association (2019). p. 363–85.

[27] Frijda NH. Emotion, cognitive structure, and action tendency. Cognition and Emotion. (1987) 1(2):115–43. 10.1080/02699938708408043

[28] Frijda NH Kuipers P ter Schure E. Relations among emotion, appraisal, and emotional action readiness. J Pers Soc Psychol. (1989) 57(2):212–28. 10.1037//0022-3514.57.2.212

[29] Elliot AJ Devine PG. On the motivational nature of cognitive dissonance: dissonance as psychological discomfort. J Pers Soc Psychol. (1994) 67(3):382. 10.1037/0022-3514.67.3.382

[30] Kavanagh DJ Andrade J May J. Imaginary relish and exquisite torture: the elaborated intrusion theory of desire. Psychol Rev. (2005) 112(2):446–67. 10.1037/0033-295x.112.2.446 15783293

[31] Janata P Peterson J Ngan C Keum B Whiteside H Ran S. Psychological and musical factors underlying engagement with unfamiliar music. Music Percept. (2018) 36(2):175–200. 10.1525/mp.2018.36.2.175

[32] Taylor IM Whiteley S Ferguson RA. Disturbance of desire-goal motivational dynamics during different exercise intensity domains. Scand J Med Sci Sports. (2022) 32(4):798–806. 10.1111/sms.14129 35037710 PMC9305115

[33] Carver CS Scheier MF. Chapter one—self-regulatory functions supporting motivated action. In: Elliot AJ, editor. Advances in motivation science. Cambridge, MA: Academic Press (2017), Vol. 4, p. 1–37.

[34] Craig AD. An interoceptive neuroanatomical perspective on feelings, energy, and effort. Behav Brain Sci. (2013) 36(6):685–6. discussion 707–26. 10.1017/s0140525(13001489 24304783

[35] Loewenstein G. Out of control: visceral influences on behavior. Organ Behav Hum Decis Process. (1996) 65(3):272–92. 10.1006/obhd.1996.0028

[36] Brehm JW Self EA. The intensity of motivation. Annu Rev Psychol. (1989) 40(1):109–31. 10.1146/annurev.ps.40.020189.000545 2648973

[37] Frijda NH Ridderinkhof KR Rietveld E. Impulsive action: emotional impulses and their control. Front Psychol. (2014) 5:1–6. 10.3389/fpsyg.2014.00518 24917835 PMC4040919

[38] Russell JA Weiss A Mendelsohn GA. Affect grid: a single-item scale of pleasure and arousal. J Pers Soc Psychol. (1989) 57:493–502. 10.1037/0022-3514.57.3.493

[39] Dickinson A Balleine B. Hedonics: the cognitive-motivational interface. In: Kringelbach ML Berridge KC, editors. Pleasures of the brain. Oxford, UK: Oxford University Press (2010). p. 74–84.

[40] Dunton GF. Ecological momentary assessment in physical activity research. Exerc Sport Sci Rev. (2017) 45(1):48. 10.1249/JES.0000000000000092 27741022 PMC5161656

[41] Hardy CJ Rejeski WJ. Not what, but how one feels: the measurement of affect during exercise. J Sport Exerc Psychol. (1989) 11:304–17. 10.1123/jsep.11.3.304

[42] Lang PJ. Behavioral treatment and bio-behavioral assessment: computer applications. In: Sidowski JB Johnson JH Williams TA, editors. Technology in mental health care delivery systems. Norwood, NJ: Ablex (1980). p. 119–37.

[43] Ekkekakis P Petruzzello SJ. Acute aerobic exercise and affect—current status, problems and prospects regarding dose-response. Sports Med. (1999) 28(5):337–74. 10.2165/00007256-199928050-00005 10593646

[44] Hall EE Ekkekakis P Petruzello SJ. The affective beneficence of vigorous exercise revisited. Br J Health Psychol. (2002) 7:47–66. 10.1348/135910702169358 14596717

[45] Svebak S Murgatroyd S. Metamotivational dominance: a multimethod validation of reversal theory constructs. J Pers Soc Psychol. (1985) 48:107–16. 10.1037/0022-3514.48.1.107

[46] Stults-Kolehmainen MA Bartholomew JB. Psychological stress impairs short-term muscular recovery from resistance exercise. Med Sci Sports Exercise. (2012) 44(11):2220–7. 10.1249/MSS.0b013e31825f67a0 22688829

[47] Stults-Kolehmainen MA Lu T Ciccolo JT Bartholomew JB Brotnow L Sinha R. Higher chronic psychological stress is associated with blunted affective responses to strenuous resistance exercise: RPE, pleasure, pain. Psychol Sport Exerc. (2016) 22:27–36. 10.1016/j.psychsport.2015.05.004

[48] Raudenbush SW Bryk AS. Hierarchical linear models: applications and data analysis methods. 2nd ed Thousand Oaks, CA: Sage (2002). 3–15.

[49] R Core Team. R: A Language and environment for statistical computing. Version 4.0 ed. (2021). p. Computer software.

[50] Bates D Mächler M Bolker B Walker S. Fitting linear mixed-effects models using lme4. J Stat Softw. (2015) 67(1):1–48. 10.18637/jss.v067.i01

[51] Kuznetsove A Brohoff PB Christensen RHB Jensen SP. Package “lmertest” [White paper]: CRAN. (2020). Available at: https://cran.r-project.org/web/packages/lmerTest/lmerTest.pdf

[52] Doyle MM Murphy TE Pisani MA Yaggi HK Jeon S Redeker NS A SAS macro for modelling periodic data using cosinor analysis. Comput Methods Programs Biomed. (2021) 209:106292. 10.1016/j.cmpb.2021.106292 34380075 PMC8435001

[53] Bittle CC Jr. Molina DJ Bartter FC. Salt sensitivity in essential hypertension as determined by the cosinor method. Hypertension. (1985) 7(6 Pt 1):989–94. 10.1161/01.hyp.7.6.989 4077226

[54] Casale R Pasqualetti P. Cosinor analysis of circadian peak expiratory flow variability in normal subjects, passive smokers, heavy smokers, patients with chronic obstructive pulmonary disease and patients with interstitial lung disease. Respiration. (1997) 64(4):251–6. 10.1159/000196682 9257358

[55] Fournier S Iten L Marques-Vidal P Boulat O Bardy D Beggah A Circadian rhythm of blood cardiac troponin T concentration. Clin Res Cardiol. (2017) 106(12):1026–32. 10.1007/s00392-017-1152-8 28856443

[56] Fekedulegn D Innes K Andrew ME Tinney-Zara C Charles LE Allison P Sleep quality and the cortisol awakening response (CAR) among law enforcement officers: the moderating role of leisure time physical activity. Psychoneuroendocrinology. (2018) 95:158–69. 10.1016/j.psyneuen.2018.05.034 29864672 PMC6401560

[57] Mills JN. Human circadian rhythms. Physiol Rev. (1966) 46(1):128–71. 10.1152/physrev.1966.46.1.128 5323500

[58] Dunlap JC Loros JJ DeCoursey PJ. Chronobiology: biological timekeeping. Sunderland, MA: Sinauer Associates (2004).

[59] Kline CE Durstine JL Davis JM Moore TA Devlin TM Zielinski MR Circadian variation in swim performance. J Appl Physiol. (2007) 102(2):641–9. 10.1152/japplphysiol.00910.2006 17095634

[60] Serin Y Tek NA. Effect of circadian rhythm on metabolic processes and the regulation of energy balance. Ann Nutr Metab. (2019) 74(4):322–30. 10.1159/000500071 31013492

[61] Clark LA Watson D Leeka J. Diurnal variation in the positive affects. Motiv Emot. (1989) 13(3):205–34. 10.1007/BF00995536

[62] Barker JL Kolar D Lazzer AS-D Keel PK. Exercise satiation: a novel theoretical conceptualization for problematic exercise observed in eating disorders. Int J Eating Disord. (2022) 55(2):176–9. 10.1002/eat.23635 34729798

[63] Boecker H Sprenger T Spilker ME Henriksen G Koppenhoefer M Wagner KJ The Runner's High: opioidergic mechanisms in the human brain. Cerebral Cortex. (2008) 18(11):2523–31. 10.1093/cercor/bhn013 18296435

[64] Ekkekakis P Hall EE Petruzzello SJ. The relationship between exercise intensity and affective responses demystified: to crack the 40-year-old nut, replace the 40-year-old nutcracker! Ann Behav Med. (2008) 35(2):136–49. 10.1007/s12160-008-9025-z 18369689

[65] Tassi P Muzet A. Sleep inertia. Sleep Med Rev. (2000) 4(4):341–53. 10.1053/smrv.2000.0098 12531174

[66] Jeong S-H Fishbein M. Predictors of multitasking with media: media factors and audience factors. Media Psychol. (2007) 10(3):364–84. 10.1080/15213260701532948

[67] Ogden J Coop N Cousins C Crump R Field L Hughes S Distraction, the desire to eat and food intake. Towards an expanded model of mindless eating. Appetite. (2013) 62:119–26. 10.1016/j.appet.2012.11.023 23219989

[68] Brietzke C Franco-Alvarenga PE Coelho-Júnior HJ Silveira R Asano RY Pires FO. Effects of carbohydrate mouth rinse on cycling time trial performance: a systematic review and meta-analysis. Sports Med. (2019) 49(1):57–66. 10.1007/s40279-018-1029-7 30488186

[69] Teff KL. Visceral nerves: vagal and sympathetic innervation. J Parenter Enteral Nutr. (2008) 32(5):569–71. 10.1177/0148607108321705 18753395

[70] Kerksick CM Arent S Schoenfeld BJ Stout JR Campbell B Wilborn CD International society of sports nutrition position stand: nutrient timing. J Int Soc Sports Nutr. (2017) 14:33. 10.1186/s12970-017-0189-4 28919842 PMC5596471

[71] Stults-Kolehmainen MA Ciccolo JT Bartholomew JB Seifert J Portman RS. Age and gender-related changes in exercise motivation among highly active individuals. Athletic Insight. (2013) 5(1):45–63. Available at: https://www.researchgate.net/publication/234111759_Age_and_Gender-related_Changes_in_Exercise_Motivation_among_Highly_Active_Individuals

[72] Stults-Kolehmainen MA Gilson TA Abolt CJ. Feelings of acceptance and intimacy among teammates predict motivation in intercollegiate sport. J Sport Behav. (2013) 36(3):320–3. Available at: https://www.researchgate.net/publication/234111733_Feelings_of_acceptance_and_intimacy_among_teammates_predict_motivation_in_intercollegiate_sport

[73] Feige K. Wesen und problematik der sportmotivation. Sportunterricht. (1976) 5:4–7.

[74] Davis WA. The two senses of desire. Philos Stud. (1984) 45(2):181–95. 10.1007/BF00372477

[75] Cheval B Radel R Neva JL Boyd LA Swinnen SP Sander D Behavioral and neural evidence of the rewarding value of exercise behaviors: a systematic review. Sports Med. (2018) 48(6):1389–404. 10.1007/s40279-018-0898-0 29556981

[76] Kaplan GB Greenblatt DJ Ehrenberg BL Goddard JE Cotreau MM Harmatz JS Dose-Dependent pharmacokinetics and psychomotor effects of caffeine in humans. J Clin Pharmacol. (1997) 37(8):693–703. 10.1002/j.1552-4604.1997.tb04356.x 9378841

[77] Duncan MJ Smith M Cook K James RS. The acute effect of a caffeine-containing energy drink on mood state, readiness to invest effort, and resistance exercise to failure. J Strength Cond Res. (2012) 26(10):2858–65. 10.1519/JSC.0b013e318241e124 22124354

[78] Tripathi R Reich SG Scorr L Guardiani E Factor SA. Lurasidone-Induced tardive syndrome. Mov Disord Clin Pract. (2019) 6(7):601–4. 10.1002/mdc3.12812 31538095 PMC6749798

[79] van de Langenberg D Vlaanderen JJ Dollé MET Rookus MA van Kerkhof LWM Vermeulen RCH. Diet, physical activity, and daylight exposure patterns in night-shift workers and day workers. Ann Work Expo Health. (2019) 63(1):9–21. 10.1093/annweh/wxy097 30551215

[80] Books C Coody LC Kauffman R Abraham S. Night shift work and its health effects on nurses. Health Care Manag (Frederick). (2020) 39(3):122–7. 10.1097/hcm.0000000000000297 32701608

[81] Nezlek JB. An introduction to multilevel modeling for social and personality psychology. Soc Personal Psychol Compass. (2008) 2(2):842–60. 10.1111/j.1751-9004.2007.00059.x

[82] Luo W Li H Baek E Chen S Lam KH Semma B. Reporting practice in multilevel modeling: a revisit after 10 years. Rev Educ Res. (2021) 91(3):311–55. 10.3102/0034654321991229

[83] Bolin JH Finch WH Stenger R. Estimation of random coefficient multilevel models in the context of small numbers of level 2 clusters. Educ Psychol Meas. (2019) 79(2):217–48. 10.1177/0013164418773494 30911191 PMC6425096

[84] Hair JF Jr Fávero LP. Multilevel modeling for longitudinal data: concepts and applications. RAUSP Manag J. (2019) 54:459–89. 10.1108/RAUSP-04-2019-0059

[85] McNeish DM Stapleton LM. The effect of small sample size on two-level model estimates: a review and illustration. Educ Psychol Rev. (2016) 28(2):295–314. 10.1007/s10648-014-9287-x

[86] Budnick CJ Santuzzi AM. Seeking reemployment in nonmetropolitan America. J Employ Couns. (2013) 50(4):146–53. 10.1002/j.2161-1920.2013.00033.x

[87] Harms R. Self-regulated learning, team learning and project performance in entrepreneurship education: learning in a lean startup environment. Technol Forecast Soc Change. (2015) 100:21–8. 10.1016/j.techfore.2015.02.007

[88] Williams DM Dunsiger S Ciccolo JT Lewis BA Albrecht AE Marcus BH. Acute affective response to a moderate-intensity exercise stimulus predicts physical activity participation 6 and 12 months later. Psychol Sport Exerc. (2008) 9(3):231–45. 10.1016/j.psychsport.2007.04.002 18496608 PMC2390920

[89] Parker M Challet E Deputte B Ract-Madoux B Faustin M Serra J. Seasonal effects on locomotor and feeding rhythms in indoor cats. J Vet Behav. (2022) 48:56–67. 10.1016/j.jveb.2021.05.005 PMC9494968 36139300

[90] Witting W Kwa I Eikelenboom P Mirmiran M Swaab DF. Alterations in the circadian rest-activity rhythm in aging and Alzheimer's disease. Biol Psychiatry. (1990) 27(6):563–72. 10.1016/0006-3223(90)90523-5 2322616

[91] Hardeman W Houghton J Lane K Jones A Naughton F. A systematic review of just-in-time adaptive interventions (JITAIs) to promote physical activity. Int J Behav Nutr Phys Act. (2019) 16(1):31. 10.1186/s12966-019-0792-7 30943983 PMC6448257

[92] Stults-Kolehmainen MA Sinha R. The effects of stress on physical activity and exercise. Sports Med. (2014) 44(1):81–121. 10.1007/s40279-013-0090-5 24030837 PMC3894304

[93] Ruissen GR Beauchamp MR Puterman E Zumbo BD Rhodes RE Hives BA Continuous-Time modeling of the bidirectional relationship between incidental affect and physical activity. Ann Behav Med. (2022) 56(12):1284–99. 10.1093/abm/kaac024 35802004 PMC9672348

[94] Stults-Kolehmainen MA Tuit K Sinha R. Lower cumulative stress is associated with better health for physically active adults in the community. Stress. (2014) 17(2):157–68. 10.3109/10253890.2013.878329 24392966 PMC4548889

[95] Stults-Kolehmainen M Filgueiras A Blacutt M. Factors linked to changes in mental health outcomes among Brazilians in quarantine due to COVID-19. medRxiv. (2021):2020.05.12.20099374. 10.1101/2020.05.12.20099374

[96] Chirazi M. Study regarding the activity of the fitness centres from the city of Iasi. Bull Trans Univ Brasov Ser IX, Sci Human Kinet. (2017) 10(1):69–75.

[97] Engelen L Chau J Young S Mackey M Jeyapalan D Bauman A. Is activity-based working impacting health, work performance and perceptions? A systematic review. Build Res Inf. (2019) 47(4):468–79. 10.1080/09613218.2018.1440958

[98] Ekkekakis P Brand R. Exercise motivation from a post-cognitivist perspective: affective-reflective theory. In: Englert C Taylor I, editors. Motivation and self-regulation in sport and exercise. New York: Routledge (2021). p. 20–40.

